# An analysis of the role of HnRNP C dysregulation in cancers

**DOI:** 10.1186/s40364-022-00366-4

**Published:** 2022-04-08

**Authors:** Liyi Mo, Lijuan Meng, Zhicheng Huang, Lan Yi, Nanyang Yang, Guoqing Li

**Affiliations:** 1 The Hengyang Key Laboratory of Cellular Stress Biology, Institute of Cytology and Genetics, Hengyang Medical School, University of South China, Hengyang, 421001 Hunan China; 2 Department of Ultrasonography, Second Affiliated Hospital, University of South China, Hengyang, 421001 Hunan China

**Keywords:** RNA-binding protein, HnRNP C, Cancers, Molecular interactions

## Abstract

Heterogeneous nuclear ribonucleoproteins C (HnRNP C) is part of the hnRNP family of RNA-binding proteins. The relationship between hnRNP C and cancers has been extensively studied, and dysregulation of hnRNP C has been found in many cancers. According to existing public data, hnRNP C could promote the maturation of new heterogeneous nuclear RNAs (hnRNA s, also referred to as pre-mRNAs) into mRNAs and could stabilize mRNAs, controlling their translation. This paper reviews the regulation and dysregulation of hnRNP C in cancers. It interacts with some cancer genes and other biological molecules, such as microRNAs (miRNAs), long noncoding RNAs (lncRNAs), and double-stranded RNAs (dsRNAs). Even directly binds to them. The effects of hnRNP C on biological processes such as alternative cleavage and polyadenylation (APA) and N6-methyladenosine (m6A) modification differ among cancers. Its main function is regulating stability and level of translation of cancer genes, and the hnRNP C is regarded as a candidate biomarker and might be valuable for prognosis evaluation.

## Introduction

Post-transcriptional processes are the main mechanisms that control gene expression in mammalian cells. RNA-binding proteins (RBPs) is a class of proteins that can mediate post-transcriptional regulation. In human cells, RBPs have their RNA-binding domains that can interact with RNAs to participate in transcriptional processes [1]. Heterogeneous nuclear ribonucleoproteins (hnRNPs) are a large family of RBPs that are rich in the human body. Studies have revealed that hnRNPs contain more than 30 proteins [2]. HnRNPs share some common functions because of their common structural features [3]. These proteins contain a highly conserved RNA-binding domain at their amino terminus, and the carboxyl end has a unique functional region that could interact with each of the RBPs [4]. They are functionally diverse and complex. They take part in the maturation of new heterogeneous nuclear RNAs (hnRNAs, also referred to as pre-mRNAs) into mRNAs and can also stabilize mRNAs, controlling their translation [5].

**Fig. 1:**
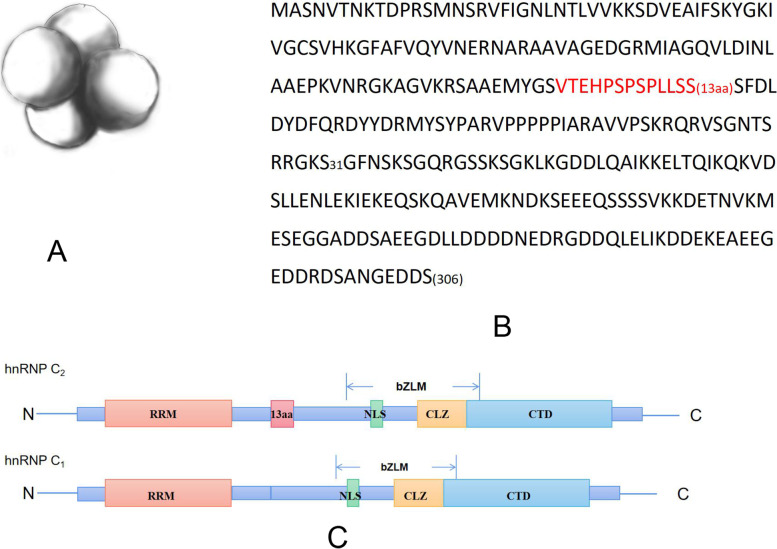
Structure of hnRNP C. **A** Tetrahedral structure of hnRNP C in electron microscope. **B** The amino acid sequence of hnRNP C_1_, the red part is the 13aa different from hnRNP C_2_.13aa:13 animo acids. **C** Functional motifs of hnRNPC_1_ and C_2_.RRM:RNA recognition motif;bZLM:Basic leucine zipper-like motif;NLS:Nuclear localization signal;CLZ: Leucine zipper-like oligomer domain;CTD:C-terminal domain

The C protein tetramer, which is called hnRNP C in this article, was identified as a core component of hnRNP particles that form on all nascent transcripts [6]. In its natural state, it is a tetramer that includes 3 C1 proteins (41 kDa) and 1 C2 protein (43 kDa) [7] (Fig. 1A). Furthermore, it has been proven that C1 and C2 come from a single coding sequence [8]. HnRNP C is located in the cell nucleus [9, 10], and binds with pre-RNA to formulate complexes, modulating splicing efficiency [11]. A study in 1986 showed that an hnRNP C antibody called 4F4 could inhibit the splicing of pre-mRNA and that 4F4 can be immunoprecipitated with a 60S splicing complex (spliceosome) that contains a C protein. Splicing of pre-mRNA was not inhibited by 4F4 deactivates or by the use of other hnRNP proteins antibodies [12]. HnRNP C could stabilize mRNA and modulate the level of translation by interacting with poly-U tracts of the 3′-uncoding region (UTR) or 5′-UTR of mRNA [13, 14]. In addition, hnRNP C is an important N6-methyladenosine (m6A) reader. The binding of these components could be facilitated by the ‘m6A-switch’ mechanism [15].

Studies on the function of hnRNP C indicate that it is necessary at the organism level but not at the cell level. Knocking out hnRNP C in mice arrests development at the egg cylinder stage [16]. HnRNP C knockout in murine stem cells only resulted in a low differentiation rate, 1and yeast, another eukaryote, also lacks this gene [17]. This means that hnRNP C1/C2 may influence the rate and/or fidelity of one or more biological processes. Indeed, many studies illuminate hnRNP C and play key roles in many human diseases. It is not clear what kind of systemic role hnRNP C plays in humans and how it is related to human disease. Herein, we reviewed the functions of hnRNP C in cancers.

## Structure

HnRNP C exhibits a unique supramolecular assembly; it is composed of four subunits: 3 C1 subunits and 1 C2 subunit [18] (Fig. 1A). In 1995, images of hnRNP C from different directions were obtained using an electron microscope [19]. Approximately 50% of the images showed that these four subunits form a plane quadrilateral. However, a triangle three-subunit structure was also shown in the images. In addition, hnRNP C has a stable structure in nature. All of these results illustrate that the macroscopic structure of hnRNP C most likely involved a tetrahedral configuration (Fig. 1A).

Isoform C2 contains 306 amino acids (aa). Isoform C1 differs from C2 in that it is missing 13 aa in the 108–120 aa region [20]; the total amino acid sequence is shown in Fig. 1B. In the N-terminus (Fig. 1C), located in the 16–87 aa region, the RNA recognition motif (RRM) is an RNA-binding domain (RBD) that is 72 aa in length [21]. Structurally, the RRM consists of four β-sheets and two α-helices (βαββαβ). It contains conserved RNP1 octameric and RNP2 hexameric sequences, and they are 30 amino acids apart [22]. The variable loops connecting the β-sheets contribute to the RNA-binding specificity of hnRNP C [23]. RRM has five binding pockets, and they can recognize uridines with an unusual 5-to-3 base selectivity gradient. Moreover, five successive fragments of U residues were screened, and binding analysis showed that this sequence constituted the binding site with high affinity (Kd = 170 nM) for hnRNP C1. It has been confirmed that full-length hnRNP C tends to bind with sequences that are rich in “U” [24].

The 155–161 aa sequence is a nuclear localization signal (NLS) (Fig. 1C), which is typically a short peptide sequence responsible for the nuclear entry of nucleophilic proteins. Typically, these sequences contain 4 ~ 8 amino acids, and the classical NLS consists of one (one-part) or two (two-part) basic amino acid chains. This result is in line with the principle that this NLS sequence is Pro-Ser-Lys-Arg-Cln-Arg-Vla [25–27].

Residues 140–214 constitute a particular domain called the basic leucine zipper-like motif (bZLM) (Fig. 1C). The functions of hnRNP C, such as stabilizing pre-RNA and mediating splicing, are dependent on the RRM and bZLM. Additionally, bZLM is a major determinant of hnRNP C’s high-affinity interaction with RNA, oligomerization and its highly synergistic RNA binding activity [17].

In the C-terminus of the bZLM, there is a 28-aa helical region (residues 180–207), which is called the leucine zipper-like oligomer domain (CLZ) (Fig. 1C). The CLZ domain of C2 and C2 itself are the binding partners of hnRNP C, and three C1 particles act as the receptors [24]. It should be noted that the specificity of RNA binding is largely mediated by the 3D structure of the protein, in which structural regions around the RNA-binding domain fine-tune the interactions of RNA proteins. The CLZ is one of these structural regions. Like many charged proteins, hnRNP C1 and C2 use alpha-helices as an oligomerization mechanism and are partially stabilized by continuous helix contacts formed between amphiphilic helix hydrophobic surfaces, and for each set of seven residues along the helix as a cyclic unit [28], a 230-nt (nucleotide) region of RNA could have four identical contacts with the RRM. C1 and C2 monomers have only one RBD, and they have to oligomerize with each other to form specific and powerful RNA interactions. This polymerization capacity is mediated by the CLZ domain [29]. This synergistic interaction between these four particles of hnRNP C is necessary to form hnRNP C tetramers to measure the length of newly formed transcripts [30]. Moreover, mutation of the CLZ domain in hnRNP C proteins results in low-affinity binding to pre-RNA [31]. The RNA site size of a single C protein tetramer is 230 to 240 nt, and three tetramers, hnRNP A, B and C, could constitute a unique 19S triangular complex that folds a single particle length of pre-RNA (700 nt). The formation of the hnRNP C triangular complex is a primary event for the assembly of other 40S hnRNP core particles in vitro and in vivo [32].

At the end of the C-terminus of hnRNP C, there is a domain called the C-terminal domain (CTD, residues 208–290) (Fig. 1C). It is worth mentioning that this domain has four phosphorylation sites that could participate in phosphorylation and dephosphorylation of hnRNP C, and studies have shown that dephosphorylation of hnRNP C proteins is necessary for their binding to some pre-RNAs [33–35]. These discoveries highlight that CTD phosphorylation and dephosphorylation are important for pre-spliceosome assembly.

Because of these functional structures, hnRNP C is able to bind to certain biomolecules and perform its functions in biological processes.

## Role of hnRNP C dysregulation in cancers

**Fig. 2:**
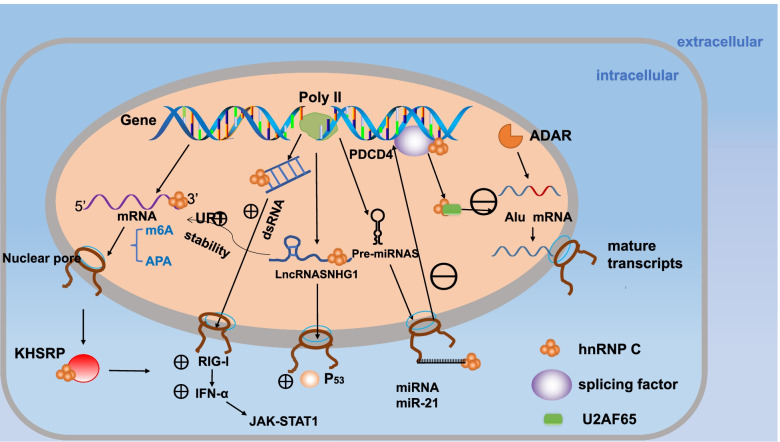
HnRNP C interacts with other molecules in cancer cells. dsRNA: double-strand RNA. RIG-I: retinoic acid-inducible gene I. IFN-α:type I interferon α. JAK: Janus Kinase. STAT1:signal transducerand activator of transcription 1. KHSRP:KH type-splicing regulatory protein. ARAD:Adenosine deaminase, RNA specific

High expression of hnRNP C has been found in many kinds of cancers, and upregulation of hnRNP C always indicates a poorer prognosis; this has been demonstrated in cancers such as breast cancer [36], glioblastoma multiforme (GBM) [37], and gastric cancer [38]. Therefore, hnRNP C is regarded as a candidate biomarker and might be valuable for prognosis evaluation. In glioma, the upregulation of hnRNP C is related to a high degree of malignancy, and clinical research has shown that the upregulation of hnRNP C might be associated with a good prognosis for glioma patients [37]. Another Kaplan–Meier survival analysis from non-small-cell lung cancer (NSCLC) showed that patients with the higher hnRNP C expression levels were predicted to have shorter survival times and to have a worse prognosis [39]. It also showed no significance in some situations, such as a study for colorectal cancer [40]. but a study for metastatic colon cancer cells showed that overexpression of hnRNP C plays a critical role in the alternative cleavage and polyadenylation (APA) profile, which has been linked to cancer progression [41]. The overview of the global functions of hnRNP C dysregulation in cancer (Table 1) shows that hnRNP C is a negative element for cancer treatment. However, it is not clear how it exerts these effects and what role it plays. Next, we will explain how it acts on different biomolecules (Fig. 2) and what regulates its expression in cancer.

**Table 1:** Role of hnRNP C regulation and dysregulation in cancers

Cancer	hnRNP C Imbalance type	Interacting molecule	Consequence
Nonsmall Cell Lung Cancer (NSCLC)	Up regulated	KHSRP protein	IFN-α-JAK-STAT1 axis activated, promoting invision and metastasis [38].
Glioblastoma multiform (GBM)	Up regulated	MiR-21	PDCD4 decreased. Promoting Migration and invision [42].
Ovarian serous cystadeno carcinoma	Up regulated	MiR-774-5p	Bcl2 levels downregulated. Promoting apoptotic [43].
Brain-trophic metastatic MDA-MB-435-LvBr2 (LvBr2) cells	UP regulated	MiR-146a	Promoting Migration and invision of LvBr2 cells [44].
Esophageal squamous cell carcinoma (ESCC)	Up regulated	LBX2-AS1 (lncRNA)	enhance the stability of ZEB1 and ZEB2 mRNAs. promoting cell EMT and migration in ESCC [45].
Oral squamous cell carcinoma (OSCC)	Up regulated	LINC00662	regulate AK4 mRNA stability. reduced the radiosensitivity of OSCC cells [46].
Breast cancer	Down regulated	dsRNA	trigger IFN response inhibit proliferation and tumor growth [35].
Colon cancer	Up regulated	URT of gene	drive CR- and UTR-APA in MTHFD1L [40].
Most of cancers	Up regulated.	As a reader Of m6A	has a potential as a biomaker.

### HnRNP C and proteins

P53 acts as a tumour suppressor in many tumour types [47]. HnRNP C was also discovered to interact with p53 by directly binding to p53 and could make p53 unstable, prevent its activation, and downregulate its protein level [48]. Moreover, it has been found that RNA can negatively regulate the hnRNP C-p53 interaction.

The expression levels of hnRNP C and KH type-splicing regulatory protein (KHSRP) in NSCLC tissues were significantly higher than those in paracancerous noncancerous tissues; KHSRP is also a pre-mRNA splicing protein. Increased expression of hnRNP C was found to be significantly associated with advanced tumour stage and metastasis The overexpression of hnRNP C significantly promoted the proliferation, migration and invasion of lung cancer cells in vitro and in vivo. Western blotting revealed that hnRNP C is a downstream protein of KHSRP and may induce the invasion and metastasis of human lung cancer cells through activation of the IFN-α-JAK-STAT1 signalling pathway (Fig. 2) [39].

### HnRNP C and microRNAs (miRNAs)

MiRNAs are a kind of noncoding RNA with a length of approximately 22 nt. They are important endogenous RNAs that can regulate gene expression and are promising candidates for biomarker development [42].

A study of GBM showed that hnRNP C could bind directly to primary miRNA and promote miRNA expression in T98G cells. When hnRNP C is silenced, miR-21 is expressed at lower levels, and programmed cell death 4 (PDCD4), which is the target gene of miR-21, is upregulated [49]. This effect on miR-21 may be due to the RNA splicing function of hnRNP C, which in turn inhibits the migration and invasion of T98G cells. Indeed, the upregulation of hnRNP C in highly aggressive U87MG cells also supported the potential value of hnRNP C as a prognostic and therapeutic marker for GBM [43]. This example shows that hnRNP C can act on miRNAs and that hnRNP C and miRNAs can interact with each other. MiR-744-5p binds to hnRNP C, and hnRNP C influences the miR-21 expression level. MiR-744-5p could lead to the downregulation of Bcl2 levels, which has pro-apoptotic effects in ovarian serous cystadenocarcinoma [44].

MDA-MB-435-LvBr2 (LvBr2) is a kind of Brain-trophic metastatic cell, hnRNP C has a high expressive in it, and virtually, miR-146a absence from brain metastases. miR-146a in LvBr2 cells could interact with hnRNP C, promoting the migration and invasion of LVBR2 cells [50]. Therefore, in human cancer cells, miRNAs downregulate hnRNP C expression. Perhaps this is a mechanism of cancer growth and metastasis. Moreover, hnRNP C shortens UTRs in mRNA APA isoforms. This shortening may improve the translational output of key genes such as cell cycle regulators by avoiding exposure to suppressor modules such as miRNAs [51]. Overall, hnRNP C directly binds and shortens UTRs, promoting the expression of miRNAs and thus influencing other cancer-related genes.

### HnRNP C and lncRNAs

Long noncoding RNAs (lncRNAs) are RNAs consisting of more than 200 nt [52], and many lncRNAs considered highly connected with cancer. Protein-lncRNA interactions play key roles in many cellular processes, such as splicing, polyadenylation, transport, stability and translation [45]. One study showed that m6A could change the local structure of mRNA and lncRNA, promoting hnRNP C binding [15]. LncRNA SNHG1 is retained in the nucleus by nucleolar binding and binds to p53-competing hnRNP C, which promotes p53-dependent apoptosis by disrupting the regulation of p53 activity by hnRNP C and upregulating p53 levels [48]. LBX2-AS1 is a lncRNA that is highly expressed in oesophageal squamous cell carcinoma (ESCC) samples. Through interacting with hnRNP C, LBX2-AS1 could enhance the stability of zinc finger E-box binding protein 1 (ZEB1) and zinc finger E-box binding protein 2 (ZEB2) mRNAs, which are the most critical epithelial-mesenchymal transition (EMT) conversion molecules, promoting ESCC cell migration [46], and it also confirmed knockdown of hnRNP C could suppress cell migration and reversed EMT progress. Similarly, LINC00662, which is overexpressed in oral squamous cell carcinoma (OSCC), recruited hnRNPC protein to increase AK4 expression. And AK4 has been demonstrated as a carcinogen. It reduces the radiosensitivity of OSCC cells [53].

These lncRNAs can effectively inhibit the binding of hnRNP C, which may explain why hnRNP C is highly expressed in carcinomas. Many cancers have been demonstrated to exhibit overexpression of hnRNP C. It is possible that Possibly hnRNP C is a downstream molecule of lncRNA.

### HnRNP C and Alu elements

In the breast cancer cell lines MCF7 and T47D, hnRNP C repression inhibited cell proliferation and tumour growth, and the supernatant from hnRNP C knockdown cells inhibited breast cancer growth [36].

The repression of hnRNP C induced the upregulation of endogenous double-stranded RNA (dsRNA), which is nonviral and known as one of the binding ligands of retinoic acid-inducible gene I (RIG-I) (gene name DDX58) [54]. However, in other tumour cell lines (MDA-MB-231 and BT549) or non-tumour MCF10A cells, hnRNP C knockout did not induce interferon (IFN) response or dsRNA accumulation in these unresponsive cells [36]. This suggests that there may be a complementary mechanism that helps retain control of the IFN response and dsRNA accumulation by compensating for the absence of hnRNP C in these cells.

This discovery of dsRNA inhibition by hnRNP C is a novel extension of the previously characterized function of hnRNP C, which binds to pre-mRNA introns and regulates RNA splicing. Endogenous dsRNA can trigger the IFN response [55]. This work is so interesting that some scientists published a comment [56]. IFN injection has been a treatment since the 1970s [57]. The activation of IFN by endogenous retroviral dsRNA was observed in hypomethylated testicular germ cell tumours, and the expression of IFN was only limited in neoplastic seminoma cells [58]. In breast cancer, endogenous nucleic acid and hnRNP C promote IFN production in cancer cells, indicating that dsRNA could at least be regulated by the hnRNP C/dsRNA axis. Moreover, the intermediate transmitter dsRNA is truly endogenous. It is a complementary mechanism product instead of a retroviral product. That is, IFN could be influenced by external factors and intracellular nucleic acids. Perhaps this finding could explain the difference in the effectiveness of IFN therapies.

These up-regulated dsRNA species are rich in the Alu sequences, which were known for harbouring hnRNP C binding sites. It could be considered that the interaction between hnRNP C and dsRNA can be seen as a part of the interaction between hnRNP C and Alu. The Alu element is a major target of the RNA editing enzyme adenosine deaminase, RNA specific (ADAR) [59]. Alu elements can act as splicing receptors, inhibit mRNA translation and cause genetic instability.

HnRNP C can prevent some splicing factors (such as U2AF65) from binding to Alu elements to protect against “Alu exonization” so that Alu elements are not at risk of abnormal incorporation into mature transcripts [60]. This phenomenon indicates that hnRNP C has a function in maintaining transcriptome stability.

However, it is unclear how hnRNP C protects against “Alu exonization”. Knockdown of hnRNP C results in the separation of the two arm exons of the Alu element and almost complete skipping of upstream replacement exons. Research has found that competition between hnRNP C and U2AF65 prevents the transcriptome from facilitating the exonization of Alu elements [60]. Deletion of hnRNP C leads to the formation of previously suppressed Alu exons, which severely disrupts transcriptional function. The inhibition of hnRNP C is Alu dependent [61]. U-bundle mutation of Alu elements mitigates hnRNP C inhibition, resulting in strong inclusion of Alu exons and skipping replacement exons. In contrast, in the hnRNP C knockdown process, the complete removal of the Alu element eliminates any regulation of the upstream optional exon. These observations are consistent with the model of dynamic processing competition between Alu exons and upstream replacement exons, and the same phenomenon occurs within the gene and downstream regions [61]. It plays a key role in facilitating the therapeutic effects of antitumour drugs such as DNA methyltransferase inhibitors and CDK4/6 inhibitors on many kinds of cancers [62, 63].

### HnRNP C and APA events

We still do not know the mechanism by which hnRNP C promotes miRNA expression and then changes the cell phenotype. APA are general mechanisms of mammalian transcriptional diversification and have recently been associated with proliferative status and cancer [64, 65].

Between normal and cancer cells, the most prominent APA profile changes have been found, and cancer cells tend to express mRNA APA isoforms [51]. APA facilitates the inclusion or exclusion of these sites (RBP sites and miRNA target sites), providing an opportunity for cells to regulate gene expression at the posttranscriptional level by affecting transcriptional stability, translation output, and subcellular localization [66]. 3′-UTR truncation of growth-promoting mRNA transcripts alleviates inhibition mediated by intrinsic miRNAs and Au-rich elements to promote facilitative translation of key genes [65]. For example, the expression of shorter mRNA subtypes of the proto-oncogene IGF2BP1/IMP-1 resulted in more oncogenic transformations than the expression of full-length annotated mRNAs [50]. In addition, this is related to the immune microenvironment in pancreatic adenocarcinoma [67].

In addition, elevated levels of hnRNP C in metastatic colon cancer cells drive coding region (CR) and UTR APA of a group of genes, including methylenetetrahydrofolate dehydrogenase (NADP+-dependent) 1-like (MTHFD1L), and these changes are closely associated with cancer progression [41]. Knockdown of hnRNP C can lengthen UTR-APA, which is shorter in colorectal carcinoma cells than in normal cells [41].

Mihaela Zavolan’s study showed that the frequency of hnRNP C binding to poly (U) bundles peaks near the poly(A) sites, and the apparent effect of hnRNP C binding sites on regulating polyadenylation decreased with increasing distance from poly(A) sites [68]. It has been reported that mRNA APA isoforms with shorter UTRs tend to be expressed in cancer cells [69]. This may be accomplished by controlling poly(A) site selection through hnRNP C to upregulate the production of full-length MTHFD1L mRNA. This inference is consistent with the observation that knockdown of hnRNP C can lengthen UTR-APA in colon cancer cells [41].

Importantly, hnRNP C could hide poly(A) sites through the tight binding of hnRNP C to the three-terminal processing sites to obscure their cleavage and polyadenylation. Both the number and length of uridine bundles contribute to the use of hnRNP C-dependent aggregation (A) sites. The downregulation of hnRNP C decreased with increasing distance between the poly(A) sites and hnRNP C binding sites [68]. When hnRNP C was knocked down, the use of intron poly(A) sites increased, which cannot be explained by alternative splicing events. When hnRNP C was knocked out, the incidence of intron site cleavage and polyadenylation also increased. However, in the terminal exon, the U-rich poly(A) sites used during hnRNP C knockout tend to be distally located. In these transcripts, hnRNP C may play the role of “blocking” the “stronger” signal at the distal end, allowing the use of the “weaker” proximal poly(A) site [70]. They also found that the intron aggregation (A) site is most likely to be deleted, which shortens the length of transcripts. This is how hnRNP C regulates APA events.

It is worth mentioning that the 3′-UTRs regulated by hnRNP C are rich in ELAV-like RBP 1 (ELAVL1) binding sites, including CD47 gene binding sites involved in the recently discovered 3′-UTR-dependent protein localization mechanism (UDPL). Indeed, hnRNP C knockout promotes the expression of the long CD47 3′-UTR [70]. This confirms that 3′-UTR-dependent proteins are hnRNP C response transcripts. CD47 protein is a hot tumour target. This also confirms the importance of hnRNP C in cancer research.

### HnRNP C and m6A

HnRNP C is one of the m6A methylation RNA regulators (“readers”) [15]. HnRNP C could alter mRNA and lncRNA in an m6A-dependent manner. M6A-related bioinformatics analysis revealed that overexpression of hnRNP C facilitates the progression of OSCC via EMT [71]. It also has multiple other functions, such as increasing differentiation in type II testicular germ cell tumours (TGCTs) [72], inducing cell death in ovarian cancer, promoting chemotherapy resistance, indicating overall survival (OS) in gastric cancer, and promoting the progression of colorectal cancer. It also showed value in diagnosis, progression and prognosis evaluation in lung adenocarcinoma, oesophageal cancer, adrenocortical carcinoma [73], urothelial carcinoma of the bladder [74], and kidney renal papillary cell carcinoma [75–77].

HnRNP C is significantly related to the OS of many kinds of cancers, such as pancreatic cancer [78], so it is an effective prognostic marker. LncRNA metastasis-associated lung adenocarcinoma transcript 1 (MALAT1), which has an m6A site, was recently shown to induce local structural changes that increase the recognition of the U5 channel and are recognized and bound by hnRNP C [79]. In addition, as an m6A regulator, hnRNP C gives rise to a malignant phenotype in pancreatic ductal adenocarcinoma (PDAC) cells by antagonizing TAF8L (antimetastatic isoform) and increasing TAF8S (prometastatic alternative splicing isoform). When an m6A mutation occurs in TAF8, the interaction between hnRNP C and the TAF8 transcript weakens, and TAF8S expression decreases [80]. This indicates that the interaction between hnRNP C and m6A mediates shearing events that affect PDAC.

The binding activity of hnRNP C regulated by the m6A switch affects the abundance and selective splicing of target RNAs. RBPs regulate RBM pathways through m6A-dependent RNA structural remodelling and provide a new direction for the study of epigenetics by RNA modification.

## Conclusions/expectations

In recent years, hnRNP C has been seen as a promising biomarker in different kinds of cancers, as a prognostic marker for cancer [81]. It influences many biological molecules to exert its effects. In most of those studies mentioned, elevated hnRNP C usually indicates a poor prognosis. However, in a few cases, hnRNP C does not show any significance [82]. This could explain why there is no perfect biomarker to estimate the prognosis of cancer patients. It has been proven to have value in most cases, so we could say it has the potential to be used in the clinic. However, with this optimistic perspective, contradicting results should also be noted. A study on GBM found that hnRNP C was positively correlated with malignancy, but when assessing the OS time of patients with high expression and low expression, hnRNP C was negatively correlated with prognosis [37]. In other research, high expression of hnRNP C indicated a poor prognosis [71]. As usual, the higher the degree of malignancy was, the worse the prognosis of the patients. This does not seem to be due to sampling error. Another study on GBM found that hnRNP C promotes migration and invasion. Overall, more data on the effect of hnRNP C on OS time would help support these conclusions.

Given that hnRNP C is located in the nucleus [83], it may not have an advantage as a clinical tumour marker. Like liver cancer biomarker AFP, a clinical biomarker should have high specificity and sensitivity and is also detectable due to its peripheral distribution. However, hnRNP C does not have these features. In the future, it is important to focus on methods for targeting hnRNP C to treat cancer.

In addition to its role in cancer, hnRNP C plays an important role in other human diseases. In the nervous system, hnRNP C binds with a 29-nt sequence in the 3′-UTR of amyloid precursor protein (APP) mRNA, whose cleavage product Aβ is highly correlated with degenerative neuropathy, such as Alzheimer’s disease, and regulates neuronal synapse growth [84–88]. These results proved its effects in stabilizing and enhancing the translation of mRNA. In addition, hnRNP C plays an important role in spinal muscular atrophy (SMA) and viral diseases such as hepatitis B virus (HBV). In addition, hnRNP C participates in ageing and regeneration. It can interact with the human telomerase holoenzyme and is related to the ability of telomerase to access telomeres [89]. After all, the maintenance of telomere length may indicate cancers instead of longer lifespan [90]. In acute promyelocytic leukaemia (APL), a novel new fusion between the HNRNPC gene and the RARG gene has been found [91]. One thing has been verified that HNRNPC-RARA is a regular genetic event instead of a random one and it is a refractory case. Indeed, hnRNP C plays an important role in many transcription-related events and maintains the stability of mRNA in normal cells. Based on the above findings, it is likely that the dysregulation of hnRNP C is a negative factor for human health.

In summary, as an RBP, hnRNP C binds with miRNA, promoting its expression. HnRNP C could bind lncRNAs, altering their effects on other molecules. HnRNP C could interact with Alu elements to prevent dsRNA from stimulating the immune response. In addition, it could interact with other genes, such as P53, to exert its effects. It also plays a splicing function, regulating APA events and m6A events, thus affecting the tumour process.

## Data Availability

The authors agree to open access for this publication.

## Abbreviations

Acute: promyelocytic leukaemia
APL: Alternative cleavage and polyadenylation
APA: Amino acids
aa: Amyloid precursor protein
APP: Adenosine deaminase, RNA specific
ADAR: Basic leucine zipper-like motif
bZLM: C-terminal domain
CTD: Coding region
CR: Double-stranded RNAs
dsRNAs: Erogeneous nuclear ribonucleoproteins C
hnRNP C: Heterogeneous nuclear RNAs
hnRNA s: Hepatitis B virus
HBV: Interferon
IFN: KH type-splicing regulatory protein
KHSRP: Leucine zipper-like oligomer domain
CLZ: Long noncoding RNAs
lncRNAs: MicroRNAs
miRNAs: Methylenetetrahydrofolate dehydrogenase 1-like
MTHFD1: Metastasis-associated lung adenocarcinoma transcript 1
MALAT1: Nuclear localization signal
NLS: Non-small-cell lung cancer
NSCLC: Oesophageal squamous cell carcinoma
ESCC: Oral squamous cell carcinoma
OSCC: Overall survival
OS: Pancreatic ductal adenocarcinoma
PDAC: RNA-binding proteins
RBPs: RNA recognition motif
RRM: RNA-binding domain
RBD: Retinoic acid-inducible gene I
RIG-I: Spinal muscular atrophy
SMA: Testicular germ cell tumours
TGCTs: Uncoding region
UTR: Zinc finger E-box binding protein 1
ZEB1: Zinc finger E-box binding protein 2: ZEB2
